# Engineering of a wireless single capacitive discharge conductivity-clamped gene electrotransfer probe for naked nucleic acid therapeutics

**DOI:** 10.1063/5.0343508

**Published:** 2026-09-28

**Authors:** Keng-Yin Lai, Stephen L. Mow, Mathumathi Manoharan, Anastasia Thiessen, Georg von Jonquieres, Jeremy L. Pinyon, Edward N. Crawford, Gary D. Housley, Amr Al Abed, Nigel H. Lovell

**Affiliations:** 1 School of Biomedical Engineering, UNSW, Kensington, NSW, Australia; 2 School of Biomedical Sciences, UNSW, Kensington, NSW, Australia; 3 School of Medical Sciences, University of Sydney, Camperdown, NSW, Australia

## Abstract

Pulsed electric field gene electrotransfer addresses the need for safe, directed “vector-free” delivery of DNA/mRNA therapeutics in deep tissue targets. We report the development of a disposable needle-type “electro-lens” probe for precision delivery of DNA/mRNA to tissues via translocation of nucleic acids into cells using focused single-pulse electric fields. We developed and manufactured a novel ganged linear bionic electrode array enhanced by incorporating integrated microfluidics to deliver naked DNA expression cassettes in a low-conductivity carrier (“conductivity-clamping,” CC). This system was packaged in a disposable wireless probe for single capacitive discharge conductivity-clamped gene electrotransfer (SCD-CC-GET), powered by an integrated capacitor with automated discharge mechanism. The electrode array was achieved by laser-etching parylene-insulated concentric stainless steel needles, with the lensing effect of the electric field tuned by varying the separation between elongated conductive elements. Our novel SCD-CC-GET probe is fully wireless and standalone, featuring integrated fluidics with minimal dead space, and an automated discharge mechanism. The probe was validated *in vitro* through HEK293 cell transfection of green fluorescent protein reporter plasmid DNA, where CC significantly expanded the transfection zone by up to 3×. *In vivo* proof of concept for deep tissue gene delivery by SCD-CC-GET was achieved in the mouse gastrocnemius muscle with long-term expression of mCherry reporter DNA. Our technology for GET is compact, low-cost, and fully wireless, with only a single point of insertion, and produces maximal electric fields at the target zone, allowing for more efficient gene therapies with minimal charge transfer, in a fine needle profile.

## I. INTRODUCTION

Electrotransfer of large DNA molecules to target tissues is achieved with pulsed electric fields driving binding of the polyanionic DNA to the cathode-facing cell membrane.^1^ While electroporation arising from pulsed electric fields includes both reversible electroporation, which can be used to drive entry of small therapeutic molecules, and irreversible electroporation, which causes cell death and finds applications in the oncology field, gene electrotransfer (GET) refers to delivery to the plasma membrane of polyanionic DNA/mRNA, which codes transgenes, and is typically of MDa size—too large to directly enter porated cells. GET relies on active internalization of the DNA by endocytotic processes, which enables trafficking to the nucleus for episomal expression,^1,2^ while mRNA released into the cytoplasm can directly drive protein translation at the ribosomes. The threshold electric field strength (voltage differential across cellular dimensions) for GET is largely determined by the voltage in the field generated by the local current flow, and hence tissue conductivity is a significant factor in efficiency of nucleic acid molecule delivery to cell membranes. We have previously shown that use of low-conductivity carriers such as sucrose for the DNA is effective at achieving suprathreshold electric field strength ( ∼ 120 V/cm) with minimal charge transfer for safe, directed gene expression in the cochlea and brain via this conductivity-clamped gene electrotransfer (CC-GET), when applied to the original “electro-lens” technology.^3,4^

The efficiency of CC-GET is notable over conventional GET approaches, which have been applied across a wide range of tissue types, including muscle, cartilage, retina, lung, cochlea, and brain.^3,5–12^ Electric pulses are most commonly delivered via plate or needle electrodes arranged in parallel. Plate electrodes establish a constant electric field but are problematic for *in vivo* applications. Needle electrode configurations, one pole per needle, can be arranged in parallel, in a grid, triangular, or hexagonal pattern, with separation distances up to 3 mm.^13–16^ These arrangements are most compatible with superficial epidermal transfections relevant to DNA vaccine applications.^17^ In these “open-field” GET applications, current pulses must be sufficient to achieve supra-threshold electric field strengths to enable DNA uptake by the cells, but constrained by the potential for tissue damage via Joule heating.^18^ Typically, voltages in the range of hundreds of volts, achieved by passing hundreds of milliamps of current, are required to achieve the supra-threshold field strengths due to the distance between electrodes.^19^ With regard to pulse type, the two main protocols include exponentially decaying pulses and square-wave pulse trains.^18,20^ The benefits of exponentially decaying pulses are that capacitive discharge circuitry has less complexity than square-wave pulse controllers, with a fixed charge based on the set voltage. However, typically GET via pulsed electric fields uses square pulses to provide flexibility for pulse height and width and inter-pulse interval. Development of the electro-lens CC-GET technology by our group overcame the limitations of open-field electrode configurations by using a linear array-based electrode configuration (e.g., “tandem” ganged 4 + 4 anode/cathode design) to control shaped electric fields as an electro-lens [Bionic array-Directed Gene Electrotransfer (BaDGE)].^3^

We have shown in an *in vitro* cell model, using reporter-encoding plasmid DNA, that BaDGE-generated electric fields adjacent to an eight-channel electrode array (derived from a cochlear implant) delineated the electro-lens—derived suprathreshold electric field, requiring minimal applied voltage/current.^3,4,21^ This was translated to an *in vivo* application, where BaDGE-based delivery of plasmid DNA encoding neurotrophins that stimulated regrowth of the cochlear nerve closed the neural gap with the cochlear implant bionic interface in deafened guinea pigs and cats.^3,9^ This was extended to proof-of-concept studies seeking to achieve precision neuromodulation of neuron subpopulations in the brain via gene augmentation relevant to treatment of focal neurological disorders such as Parkinson's disease. In those studies, CC-GET delivery of a plasmid DNA cocktail encoding a genetically encoded Ca^2+^ reporter and a channelrhodopsin enabled selective regulation of excitability measured by Ca^2+^ signal dynamics.^3^

While initial development of the BaDGE system was performed using an eight-channel cochlear implant electrode array,^4,9,22^ we determined that the electro-lens derived suprathreshold electric field tuning could be achieved in a two-channel longitudinally extended anode and cathode configuration, which, with integrated fluidics for DNA/mRNA delivery and conductivity-clamping, would be ideal for deep tissue gene delivery targeting applications aligned with focal neurological disorders^23^ and vector-free DNA/RNA vaccine applications.^24^ This manuscript describes the biomedical engineering required to achieve this new focal gene electrotransfer technology, which addresses the limitations of current open-field electrode configurations, in which multiple invasive needle/plate electrodes are required, leading to high charge transfer requirements that impact tissue integrity and generate pain sensation. The present study reflects iterative development of a compact probe for conducting GET, with spatial control of DNA/mRNA solution dispersion relative to the pulsed electric field. This enabled a single point of insertion into the target tissue using a compact gene delivery probe with wireless charging and pulse delivery. The primary focus of this study is on the engineering of this novel nucleic acid delivery platform, centered on the development of the electro-lens technology. To this end, we report on the design and construction, with *in vitro* validation of GET of naked reporter DNA to adherent HEK293 cells, as well as *in vivo* validation of DNA delivery to mouse hindlimb skeletal muscle.

Table I compares the operational and clinical limitations of existing open-field gene electrotransfer with the specific hardware, biophysical, and delivery advantages offered by the proposed BaDGE single capacitive discharge conductivity-clamped gene electrotransfer (SCD-CC-GET) platform.

**TABLE I.** Comparison of the proposed BaDGE SCD-CC-GET platform with conventional clinical GET technologies.

Comparison category	Conventional “open-field” electroporation (e.g., TriGrid, CELLECTRA)^25^	Proposed BaDGE SCD-CC-GET platform
Needle/electrode architecture invasiveness	Highly invasive, requires 2–5 penetrating needle electrodes arranged in parallel, rectangular, or diamond formations	Minimally invasive, requires only a single insertion, with both electrodes arranged concentrically
Pulse generation hardware	Relies on bulky external tethered stimulators and pulse generators	Fully wireless and standalone, powered by internal discharge mechanism integrated into disposable syringe hub
Typical pulse settings	Square-wave pulses, e.g., 3 × 52 ms at 0.5 A, 4 × 52 ms at 0.2 A, 2 × 60 ms at 106 V, 2 × 60 ms at 0.4 A	Single exponentially decaying capacitive discharge pulse (e.g., via a 2.2 *μ*F capacitor)
Electric field localization	“Open-field” current spread between parallel widely spaced electrodes (typically ∼4 mm separation)	“Close-field” localized compression via insulated tunable “electro-lens” gap (∼1 mm or less) between linear electrodes
Carrier solution/conductivity	Operates in highly conductive physiological saline, requiring higher currents to achieve target electric field strengths	Utilizes “conductivity-clamping,” where low-conductivity nonionic carrier (e.g., 10% sucrose^3^) decreases local conductivity and artificially amplify the focal electric field
Charge transfer/safety	Transfers tens of millicoulombs of electrical charge which can induce Joule heating and irreversible cellular electroporation	Operates with a fraction of the charge transfer (microcoulombs rather than millicoulombs), virtually eliminating Joule heating and improving tissue viability

## II. CONCEPT IDEATION

To evaluate the dynamic control of electro-lens focusing for GET using a two-electrode configuration, we employed an *in silico* finite element computational model (COMSOL AB, Sweden). Within the physics settings of the computational model, we implemented the following stationary form of Maxwell's equations

−∇·σ∇V=0 (1)

over a tetrahedral finite element mesh, with the following boundaries conditions

n·J=0 Internal, (2a)

V=0 Ground electrode, (2b)

$$V=V_{\text{stim}}\text{ Active electrode},$$ (2c)

where *J* is the current flux at the boundary and $V_{\text{stim}}$ is the applied voltage. The conductivity, σ, was set to 1 S/m. The maximum triangular mesh element size of the boundaries representing the electrodes and the inter-electrode gap was set to 10 μm and that of the tetrahedral mesh to 100 μm.

The models demonstrated that a two-electrode electro-lens can generate sufficient field strength for GET, comparable to that of a tandem configuration eight-electrode array as shown in Fig. 1. These simulations also predicted that the size of the field can be shaped (electro-lens focusing) by varying the distance between electrodes relative to the length and diameter of the electrodes (Fig. 2). They also established that while increasing the electro-lens gap to 1 mm progressively expands the suprathreshold electric field boundary, larger separation distances (e.g., 4 mm) beyond this lead to partitioning into discontinuous areas around each electrode, reducing the overall transfection zone. This modeling confirms the spatial control of the critical electric field previously demonstrated *in vitro* using monolayers of HEK293 cells transfected with green fluorescent protein (GFP) reporter plasmid DNA.^3^

**FIG. 1.**
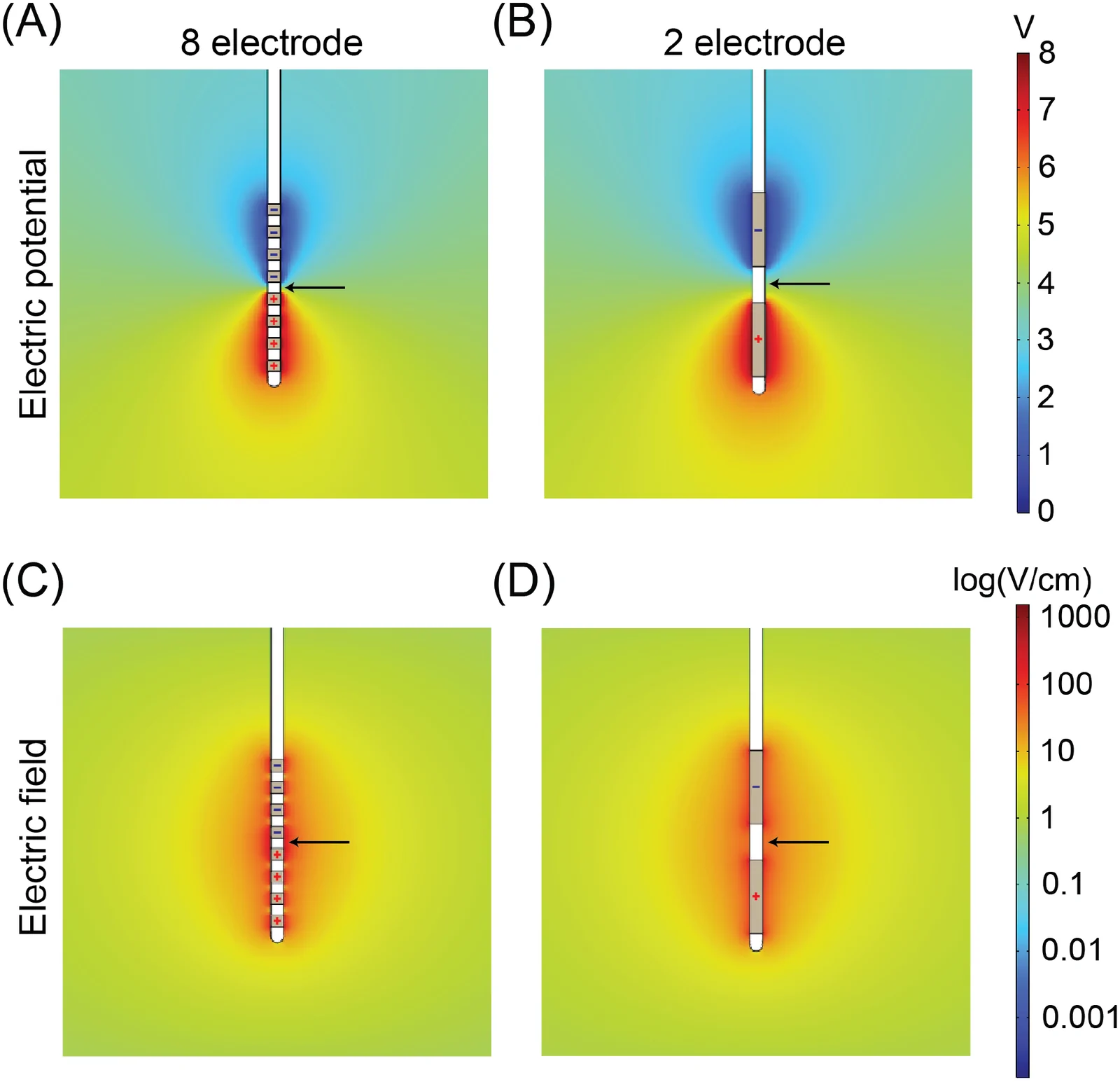
COMSOL modeling of BaDGE electro-lens field mapping comparing a tandem-ganged 4 + 4 electrode array (350 μm elements, 350 μm separation within anode and cathode arrays, with 350 μm electro-lens gap (tuning element shown by arrowhead) between anode and cathode zones) vs 2 elongated primary electrodes (2 mm anode/cathode; 1 mm electro-lens gap). This modeling indicates comparability between use of two longitudinally extended electrode elements with a separating electro-lens tuning gap, and the ganged smaller circular electrode array elements.

**FIG. 2.**
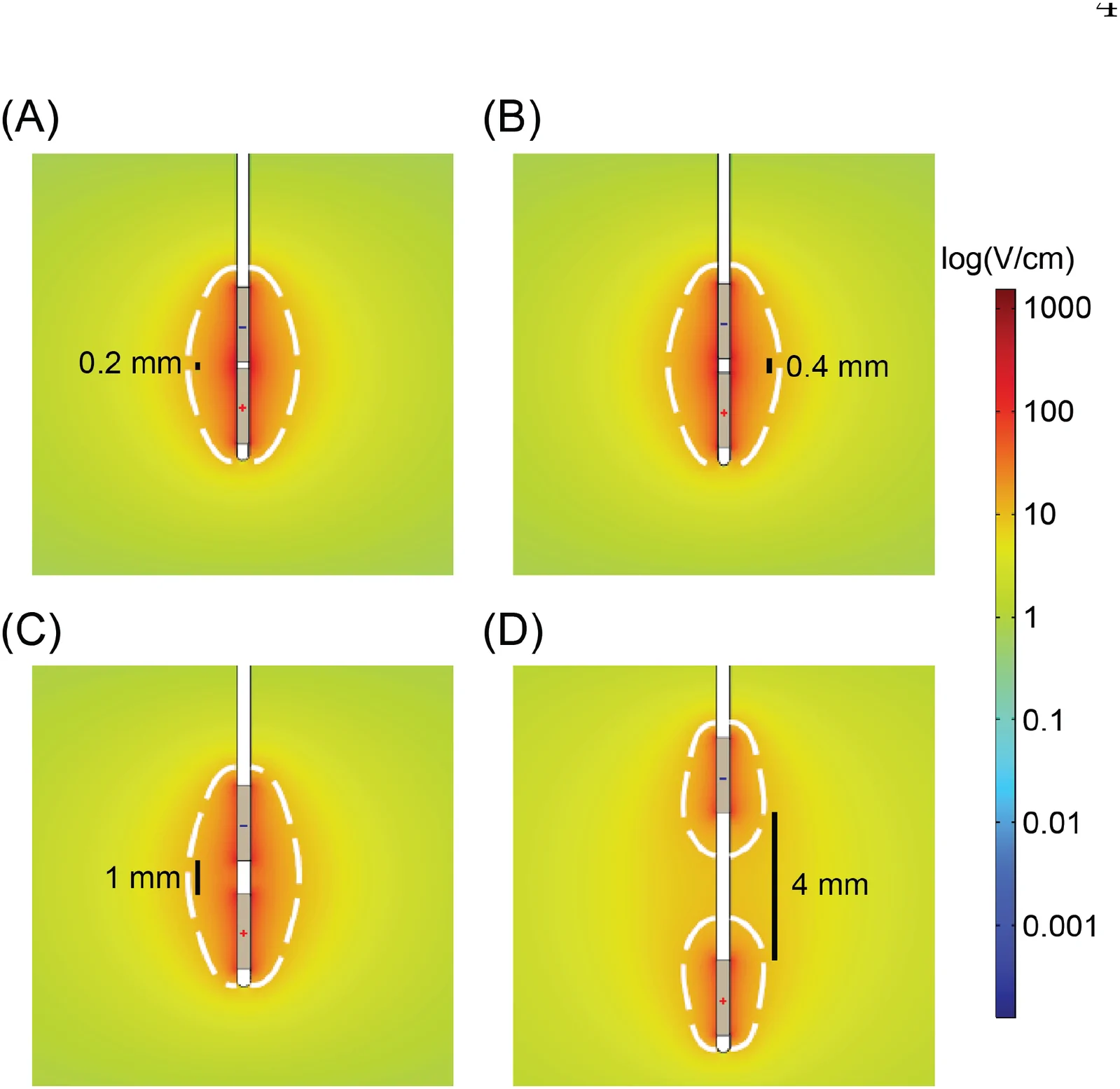
COMSOL modeling of effect of BaDGE electro-lens gap on gene electrotransfer threshold boundary. Comparing simulated electric fields for various 2 electrode anode/cathode separation distances. Electro-lens gap distances of (a) 0.2, (b) 0.4, (c) 1, and (d) 4 mm indicated by vertical black bars. Dashed white boundaries compare the encapsulated area at 10 V/cm (arbitrary, for the purpose of illustration), showing a gradual lengthening of the suprathreshold zone up to 1 mm, but a subsequent partitioning of the zone at an increasing distance between the electrodes (e.g., 4 mm), which would result in a decrease in transfection efficiency.

The rigid probe design inherent to the two-channel linear electrode configuration is ideal for penetrating deep tissues such as muscle and the brain, with the ability to customize the transfection volume via electro-lens focusing to achieve GET matched to the gene expression spatial requirements. Furthermore, by arranging the two electrodes on two concentric needles, fluid delivery can be achieved either through the inner needle or between the needles. Figure 3 shows a conceptual diagram of how the probe may eventually look and operate. The probe would be a wireless attachment that screws onto a standard syringe (a). After being charged via an external wireless charger (b), the needle element of the probe would be inserted into the patient with the DNA/mRNA in carrier solution being delivered using the syringe plunger (c). On completion of the delivery, the probe would then discharge via the internal discharge mechanism, producing the necessary electric fields for GET (d). The compact and wireless nature of this probe lends itself to several possible applications (e) including DNA vaccine delivery to muscle tissue, treatment of cortical structures in the brain, targeting of tumors located within organs such as the liver, and applications in cell cultures such as HEK293 monolayers, organoids, and organotypic tissue cultures.

**FIG. 3.**
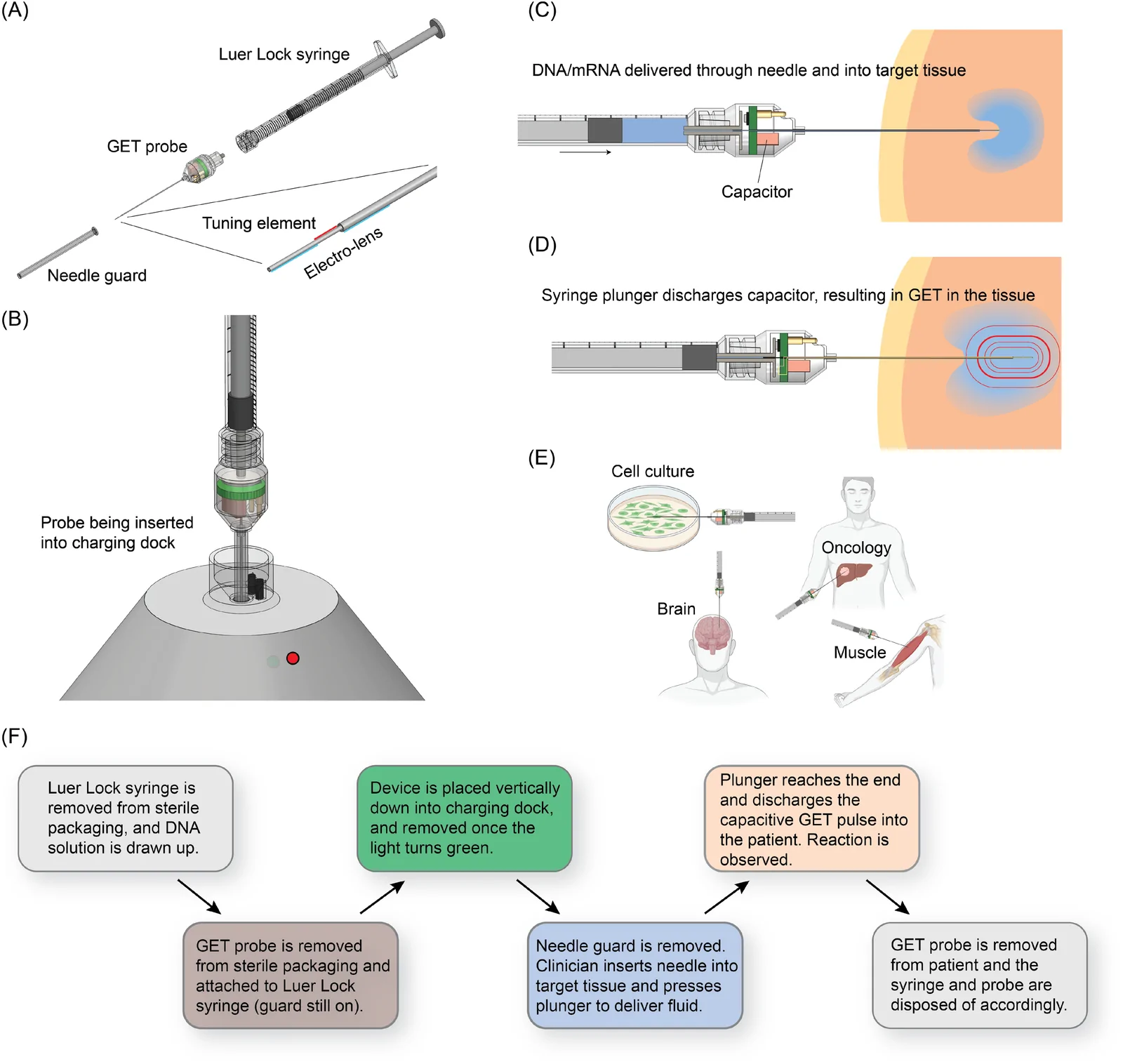
Conceptual representation of a compact wireless probe for naked DNA/mRNA GET. (a) Mockup of the GET probe, shown to attach to a Luer Lock-type syringe. (b) The integrated capacitor of the GET probe within the hub of the GET probe is pre‐charged to a set voltage using an external charging dock. (c) DNA/mRNA within a low-conductivity vehicle (10% sucrose) is delivered into the target tissue through the electro-lens, bypassing the internal electronics. (d) Syringe plunger depression triggers capacitor discharge, producing a single electric field pulse in the tissue, causing the polyanionic nucleic acid molecules to adhere to target cell membranes for translocation to the cytosol–nucleus. Transfection volume can be predicted according to the field contours (red lines). (e) The tunable instantaneous delivery of naked DNA/mRNA opens a pathway for highly personalized nucleic acid therapeutics including tumor targeting, brain tissue, and muscle DNA vaccines, as well as *ex vivo* transfection for cell-based therapies. (f) Flow chart summarizing the proposed operating sequence of the BaDGE GET probe in eventual clinical settings.

## III. FABRICATION OF BaDGE GET PROBES UTILIZING CYLINDRICAL PLATINUM ELECTRODES

An initial proof-of-concept BaDGE GET probe was created en route to the final design, with a focus on demonstrating the feasibility of combined fluidics and electric pulse delivery on a single probe (prior to considering wireless discharge of an integrated capacitor). The basic design of this probe is shown in Fig. 4(a), consisting of two 2 mm platinum tube electrodes attached to a 34-gauge INViSIBLE needle (TSK Laboratory Europe, Netherlands) with wires attached for connection with an external stimulator to deliver the electric pulses.

**FIG. 4.**
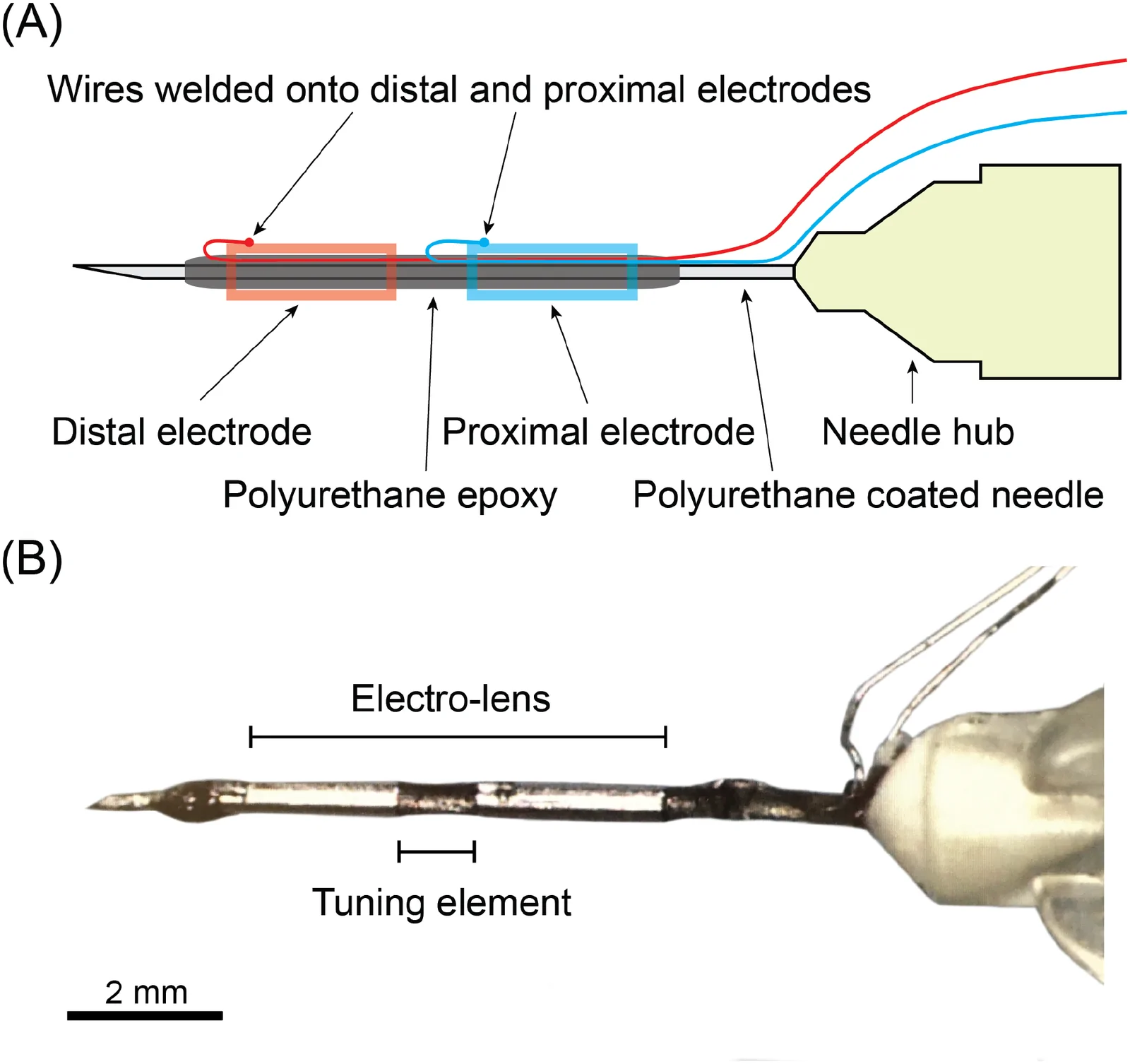
Platinum cylinder “electro-lens” elements in the BaDGE GET probe. (a) Schematic of the tethered Pt/Ir tube BaDGE probe. (b) Constructed tethered Pt/Ir tube electro-lens with electrode separation reflecting the tuning element for focusing the electric field shape.

The wires (Pt/Ir alloy with a diameter of 100 μm) were welded onto 2 mm Pt/Ir tubes (with an outer diameter of 400 μm) using an Optec JK-400FL laser system (Optec S.A., Belgium), at a point 300 μm from the end of each tube. INViSIBLE needles (200 μm outer diameter, length of 9 mm) were coated with a PUC polyurethane conformal coating (Electrolube, UK) such that the needle would be fully insulated on the external surface, with a total thickness averaging 288 ± 7 μm (n = 5 test probes). The Pt/Ir tubes (with attached wires) were then threaded onto the insulated needle with a separation distance of 1 mm between them (the electro-lens tuning element). Finally, black polyurethane encapsulation resin UR5528 (Electrolube, UK) was applied under and around both Pt/Ir tubes in order to fix them in place and to further insulate the electrodes from the needle. Figure 4(b) shows an example of a completed GET probe. This design underpins the GET of reporter plasmid DNAs into mouse hindlimb skeletal muscle.^24^ A limitation of this design was the complexity of the assembly (in particular, preserving the insulation of the wires while sheathing the electrodes onto the needle), and the difficulty in achieving perfect separation and alignment between the two electrodes. Hence, in our eventual design of the electro-lens, we advanced the concept by etching elongated electrode surfaces separated by an insulated tuning element on stainless steel (SS) concentric needles to achieve finer manufacturing tolerances for electro-lens production, facilitating ease of tissue penetration, while preserving flexibility in electrode lengths and separation distances inherent in creating the tunable focused electric field for GET. The electrical properties and suitability of SS as an electrode material instead of Pt/Ir for electro-lens production are assessed in the characterization studies.

## IV. FABRICATION OF WIRELESS CONCENTRIC NEEDLE BaDGE PROBES

Having demonstrated the feasibility of a combined fluid and electric pulse delivery probe, we moved toward improving the reliability and consistency of the design of the linear electrode array, and transitioned from the tethering external stimulator to wireless integrated capacitor charging. To achieve compactness and reduce cost, the linear electrode array changed from consisting of separate and isolated Pt/Ir tubes to being a pair of insulated concentrically aligned stainless steel (SS) needles with laser-etched electrode surfaces, which serve both functions of fluid and pulse delivery while minimizing probe diameter transitions. To achieve wireless operation, a needle hub was designed to incorporate an onboard capacitor that could be charged via contact points in a docking station, and then discharged via a switch. Such a GET probe would also be low-cost (due to the material choice and simplicity of electronics) and disposable, lending itself to applications such as naked DNA/mRNA vaccine immunotherapies in the field.

### A. Design and construction of needle-type BaDGE GET electrode array

The novel advance in the design and construction of the linear electrode array was a technique in which the “electrodes” would be etched out from two concentric SS needles insulated from each other and attached onto either side of a printed circuit board (PCB) small enough to be integrated into a needle hub. This design ensured a more consistent electrode separation, as well as a reduction in polyurethane resin and elimination of Pt/Ir wires, compared to the previous tethered Pt/Ir tube BaDGE probe design. Figure 5 shows a flow chart of the construction process. The 304 alloy SS needles were custom manufactured (VitaNeedle, USA), with dimensions of ∅ 0.184/0.083 mm × 51.5 mm (including 0.5 mm of cutting edge) for the 34-gauge inner needle, and ∅ 0.362/0.267 mm × 45 mm for the 28-gauge outer needle. SS annuli with dimensions of ∅ 1.2/0.362 mm and a thickness of 0.15 mm were cut from a tube using an Nd:YAG laser (ACI Laser GmbH, Germany) and welded onto the end of the outer needles in order to provide a surface on which to solder the needles onto PCBs [Fig. 5(a)]. The welding was performed using an Optec JK-400FL laser system (Optec S.A., Belgium) through a welding lens with the needles and annuli fixed inside a helium flooded chamber. Both the outer needles and inner needles were then suspended on inhouse-built tooling using monofilament nylon suture, which was then placed into a Diener Femto plasma etcher (Diener Electronic, Germany) in order to roughen the surfaces prior to application of the insulating layers [Fig. 5(b)]. The needles underwent surface roughening by reactive ion etching for 30 min with a plasma ratio of 3:1 tetrafluromethane:oxygen.

**FIG. 5.**
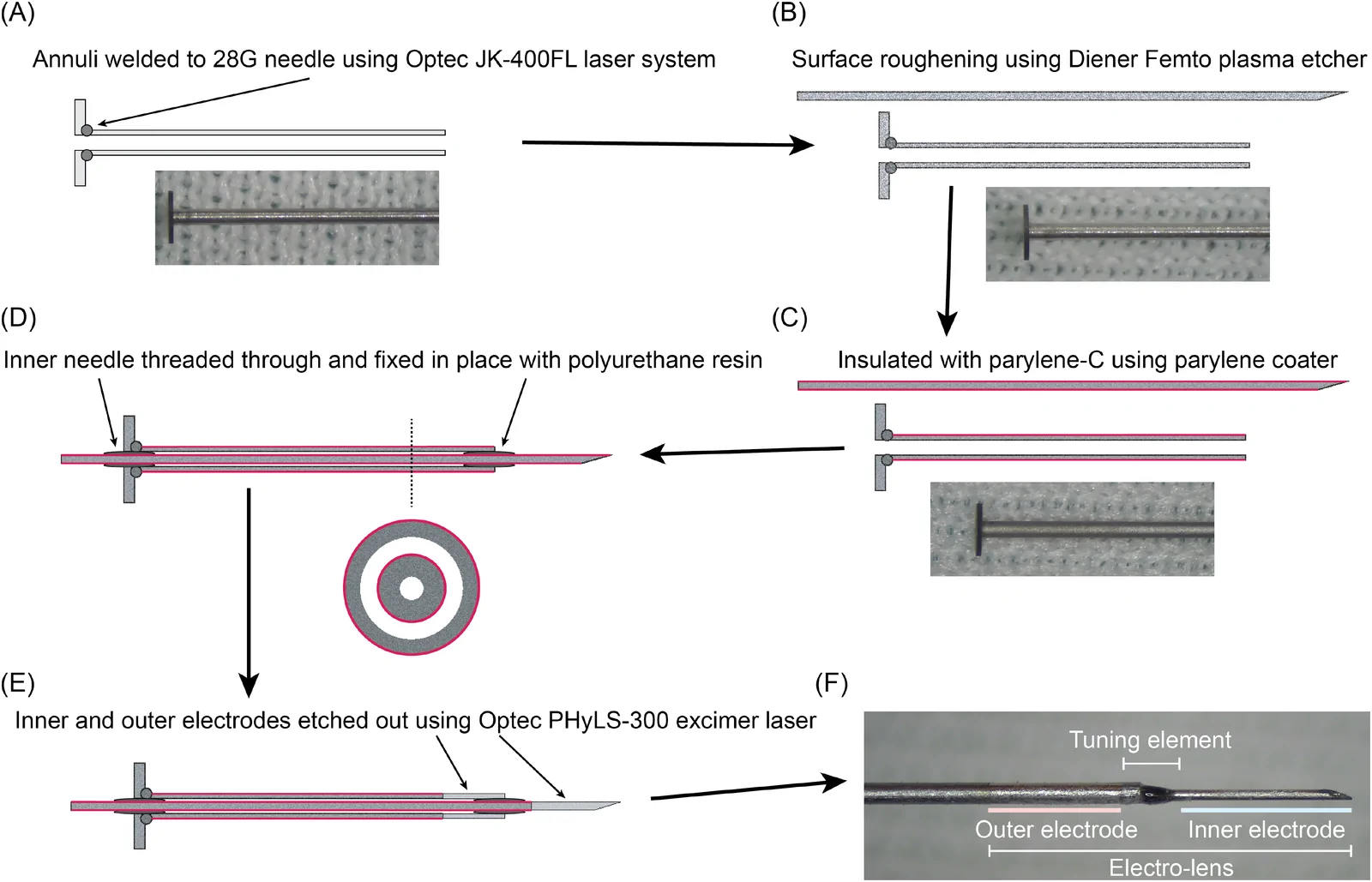
Work flow of concentric needle GET probe construction. (a) Welding annuli onto proximal (needle hub end) of outer needle. (b) Plasma-etching of stainless steel (SS) needles to improve parylene-C insulation adhesion [note difference in needle surface texture in image compared with (a)]. (c) Insulating needles with parylene-C by vapor deposition to ∼ 5 μm (red). (d) Assembling the insulated inner needle within the outer needle (inset shows parylene-C coated surfaces in red). (e) Precision laser-etching removal of parylene-C insulation around the inner (tip region) and outer needle proximal to the insulated tuning element, forming longitudinally extended (2 mm) SS electrodes. (f) Completed BaDGE GET electro-lens. Images show outer needle = 28-gauge thin-walled (362/266 μm outer/inner diameters) and inner needle = 34-gauge (184/83 μm outer/inner diameters).

The needles were then insulated using parylene-C (LabTop 3000 Parylene coater, Para Tech Coating, USA) at the NSW node of the Australian National Fabrication Facility (ANFF). Parylene-C dimer in powder form (5 g) was placed inside the vaporizer, equating to 5 μm of vapor-deposited parylene-C coating on the needles (conferring ∼ 1.1 kV dielectric breakdown). The deposition stage lasted for 30 min, with a total time including sublimation and pyrolysis of 1.5 h. Figure 5(c) shows the thin and highly conformal parylene-C coating on the needles. The next step consisted of threading the inner needles through the outer needles, such that the inner needle protruded from the outer needle by 3 mm (plus cutting edge) at the cutting edge end [Fig. 5(d)]. The protrusion at the back end (the annulus end) was also 3 mm. A small amount of UR5528 polyurethane resin (Electrolube, UK) was applied at either end of the outer needles to fix the inner needles into place. Finally, the parylene-C coating was etched out of the inner and outer needles at the cutting edge end using an Optec PHyLS-300 laser system (Optec S.A., Belgium) with a 193 nm excimer laser [Fig. 5(e)], pulsed at 50 Hz with 100 shots at an energy output of 4–5 mJ. 2 mm of insulation was precisely etched away from each needle, resulting in an accurate 1 mm separation distance (tuning element), matching the optimal dimensions predicted by the *in silico* modeling. The completed needle pairs, shown in Fig. 5(f), were then soldered onto PCBs using SS solder, and housed in inhouse 3D printed hubs containing the electronics required to form a fully functional BaDGE GET probe. The evolution of the electronics and hub designs are detailed in Sec. IV B.

### B. Design and construction of internal electronics and housing for single capacitive discharge BaDGE GET probe

The combination of very high electric field compression achieved by the electro-lens along with conductivity-clamping (after Ref. 3) meant that the charge transfer for suprathreshold field strengths sufficient to drive gene expression was minimal and compatible with a single pulse from a miniature capacitor.^3,26^ The design requirements for our new GET probe were thus (1) the ability to deliver a single exponentially decaying electric pulse via the two exposed electrodes, without the use of an external pulse generator or additional actions by the user—this would be achieved by housing a capacitor that could be charged up prior to gene delivery, and then be discharged into the tissue after gene delivery via a fully self-contained automated mechanism; (2) minimal dead space (and variance), preferably to below 50 *μ*l, to reduce wastage of DNA/mRNA solution and ensure consistent dosage; and (3) consistent and reliable electrical discharges that are not negatively affected by the proximity of charged fluid during delivery of the solution.

The capacitor chosen for this probe was a 2.2 μF multilayer ceramic capacitor (TDK, Japan) with a voltage rating of 450 V, the maximum voltage for this capacitor type. Typically, in the GET experiments performed with this probe, the capacitor would be charged up to 120–200 V, while the discharge time constant would depend on the resistance of the tissue and the electrode–electrolyte interface (controlled by “conductivity-clamping”^3^). All PCBs were developed using a software called KiCad (open source), and manufactured by JLCPCB (JLCPCB, China). The components mounted on the top side of the PCB (spring and receptacle) were manufactured by MillMax (MillMax, USA). The hub pieces in which the circuitry was enclosed were designed primarily in SolidWorks (Dassault Systemes, France), and printed in 405 nm UV sensitive resin using an LCD-based SLA 3D printer, the Anycubic Photon Mono (Anycubic, China). In Secs. IV B 1 and IV B 2, we detail the design of the discharge mechanism and associated circuitry.

#### 1. Automated BaDGE single capacitive discharge mechanism

The design concept is illustrated in Figs. 6(a) and 6(b). The BaDGE device consists of three main assemblies: a syringe sleeve, copper switch, and needle hub. The syringe is clipped into the sleeve, which incorporates a mechanism capable of detecting when the syringe has been fully depressed. The detection mechanism (a trigger) is situated at the base of the syringe plunger clip-on. This enables accurate detection of the end-of-stroke. The capacitor is discharged by closing the circuit when the plunger clip-on presses the trigger into the copper switch. This is visualized in Fig. 6(b). The copper switch consists of copper pads and a copper spring, which is pressed down via the trigger upon depression of the plunger. The trigger is able to travel up and down along a mechanical guideway. To implement all of the above, the entire mechanism is integrated into a custom syringe sleeve that clips onto any standard syringe, into which the hub (housing the needle electrode array and PCB) is then inserted.

**FIG. 6.**
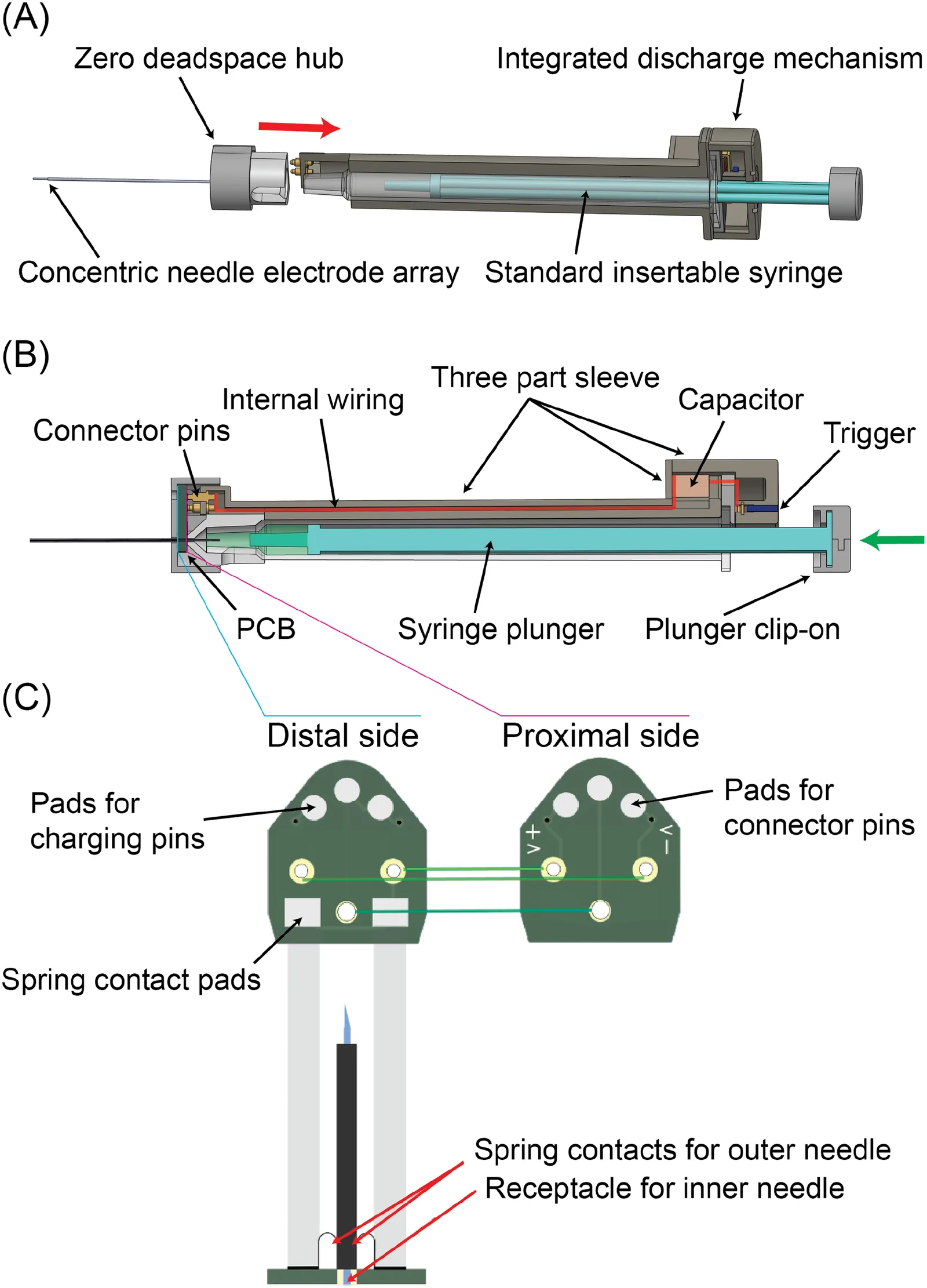
Design and construction of syringe housing integrating the concentric needle array, automated discharge mechanism, and associated circuitry into a snap-fit assembly with small footprint and minimal dead space. (a) A standard syringe is placed into a sleeve piece containing the discharge mechanism. The zero-dead space hub housing the PCB and concentric needle array is then inserted into the sleeve, connecting the PCB electrically to the discharge mechanism above. (b) When the syringe plunger is depressed by the user, fluid is delivered through the needles (shown in green). At the end of its travel, the plunger clip-on will depress the trigger into the copper switch, thus closing the switch. (c) A circuit diagram showing how the pads on the proximal side of the PCB connect the discharge mechanism in the sleeve above to the inner and outer needles. The inner and outer needles are slotted into a receptacle and spring contacts, respectively. On the distal side of the PCB are pads for charging via the charger.

#### 2. Circuit design

The circuit design provides electrical connectivity between the switch assembly at the top end of the sleeve and the needle electrodes and needle hub at the lower end of the sleeve. The schematic in Fig. 6(c) details all required interfaces and highlights critical connection points. The electrical connection between the upper and lower sections of the sleeve was achieved using a combination of copper tape and enameled wire. Connectivity to the PCB was established via enameled wires soldered to pogo pins, which make contact with designated PCB pads. On the other side of the PCB, the charging pins align with three contact points, allowing for safe and reliable charging of the device.

The two concentric needle electrodes must be connected separately. The outer electrode is connected via springs which snap onto the metal. The second connection from the inner needle to the PCB is made by insertion into a press-fit part (receptacle) on the PCB. Therefore, the parylene-C coating of the needle must first be removed at this connection point, prior to its connection. The final outcome can be seen in Fig. 6(c). The square pads designate where the springs are soldered. Additionally, test pads were implemented in order to monitor the circuit via an oscilloscope. On one side of the PCB, the pogo pins were pressed against the PCB surface and on the other side the charging pins were pressed against the pads. This design enabled the needle electrodes (in its hub) to remain separate from the rest of the device, which improved handling during fabrication, since the electrodes must remain sterile.

Figure 7 shows photographs of the completed automated BaDGE device, with full assembly in A, a close-up of the needle hub attachment in B, and copper switch discharge mechanism in C and D.

**FIG. 7.**
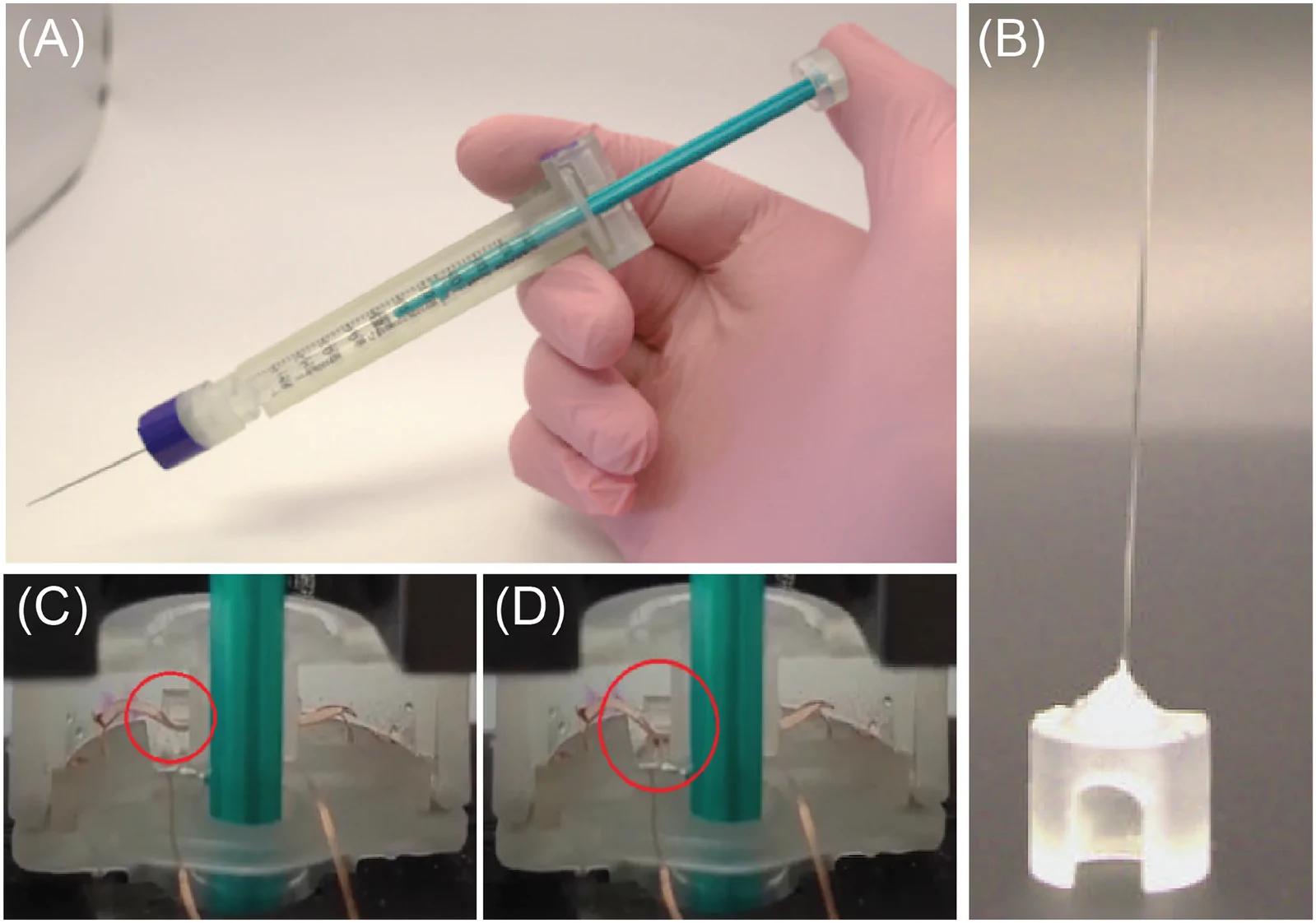
Photographs of a completed automated BaDGE device. (a) Fully assembled device can be handled with one hand, where the completion of the plunger movement fully delivers the fluid in the syringe and triggers the discharge. (b) Close-up of the zero-dead space hub with concentric needle array attached flush into the hub. The PCB component is not shown in this image. (c) Zoomed-in image of the discharge mechanism in which the copper switch has not been depressed, vs (d) where the copper switch has been pressed together, causing the circuit to be completed for discharge of the capacitor.

## V. ENGINEERING CHARACTERIZATION

A number of characterization studies were conducted on the new wireless concentric SS needle GET probe to assess their operational reliability against the design criteria. The first study was to determine the amount of dead space volume within the probe, as this would affect the volume of solution required to be loaded into the syringes. Second, tests were conducted to determine the consistency of the capacitive discharges and their resulting waveforms, as an indication of the reliability of the onboard automated discharge mechanism. Finally, a study was conducted to compare the use of SS as an electrode material compared with Pt/Ir with respect to voltage losses across the electrode–electrolyte interfaces.

### A. Fluid delivery

Dead space volume was quantified for four batches of devices (with 5–10 devices per batch) through weighing of the syringe–device assemblies before and after fluid delivery. While the initial pilot batch had a mean dead space of 24.2 μl ± 13.3 (n = 5), batches 2–4 reported dead spaces of 12.8 μl ± 10.6 (n = 10), 10.7 μl ± 6.1 (n = 10), and 5.1 μl ± 3.7 (n = 10), respectively, representing a substantial reduction in both average volume and standard deviation compared with the pilot batch. Across all measurements, the combined system yielded a population mean dead space of 13.2 μl ± 4.01. To independently assess syringe retention, two syringes were subjected to repeated weighing cycles (five per syringe), resulting in a measured mean dead space of 13.3 μl ± 7.06. While limited by sample size, these measurements fall within a plausible range when compared with what is known of syringe dead spaces. Subtracting the estimated syringe contribution from the total measured values indicates that the device in combination with low-dead space syringes achieves a configuration that is effectively near-zero in device-associated dead space.

### B. Electrical discharge properties

The capacitor discharge profiles upon switch closure were obtained by monitoring the voltage across the capacitor via test pads using an oscilloscope (DS1074, Rigol Technologies, China). Data were collected onto a USB storage device and subsequently processed using a Python script. This circuitry allows for the repeated charging and discharging of the capacitor across two electrodes. For each test, a 1 k Ω resistor coupled to the BaDGE probe electrodes was used to model resistance in tissue. For a 2.2 μF capacitor, this equated to a theoretical 2.2 ms decay constant ( τ = RC). The capacitive discharge behavior of five switches (with n = 9, 11, 11, 11, 12 trials each) was assessed under identical test conditions, with the capacitors charged to approximately 30 V using an external power supply. The peak voltage levels were correct across all trials, and the time constants of the discharge aligned well with theoretical expectations in the majority of trials across all switches. Tiny pre-discharge bouncing was observed across switches, but this did not distort the ultimate discharge profiles. The switch press success rate was determined by evaluating the number of successful discharges over the total number of activations for each switch configuration. Overall, the average success rate in achieving a complete and clean discharge across the 54 trials from 5 different switches was 94.14%.

### C. Comparison of electrode materials

A key question we addressed was whether the use of SS was suitable as an electrode material for the wireless concentric needle BaDGE GET probes, compared with use of Pt/Ir; given the anticipated voltage loss due to electrode–electrolyte impedance. Electrical impedance spectroscopy (EIS) was performed on n = 4 Pt/Ir tube probes and n = 4 concentric SS needle probes. Note that the electrode surface areas were not identical (the Pt/Ir tubes had a diameter of 400 μm, while the concentric SS needle probe had diameters of 184 and 362 μm for distal and proximal electrode surfaces). Results were normalized to the respective electrode surface areas (in the concentric SS case the average area of the distal and proximal electrodes is used) to account for this. The GET probes were suspended in 100 ml of 0.9% saline, and the spectroscopy was performed using a CHI604E electrochemical analyzer (CH Instruments, USA), set to obtain 12 points per decade between 1 and 100 000 Hz, with a voltage amplitude of 0.01 V. Figure 8 shows the mean (with standard error) impedance (normalized to electrode surface areas) across the 1–10 000 Hz range for each probe type.

**FIG. 8.**
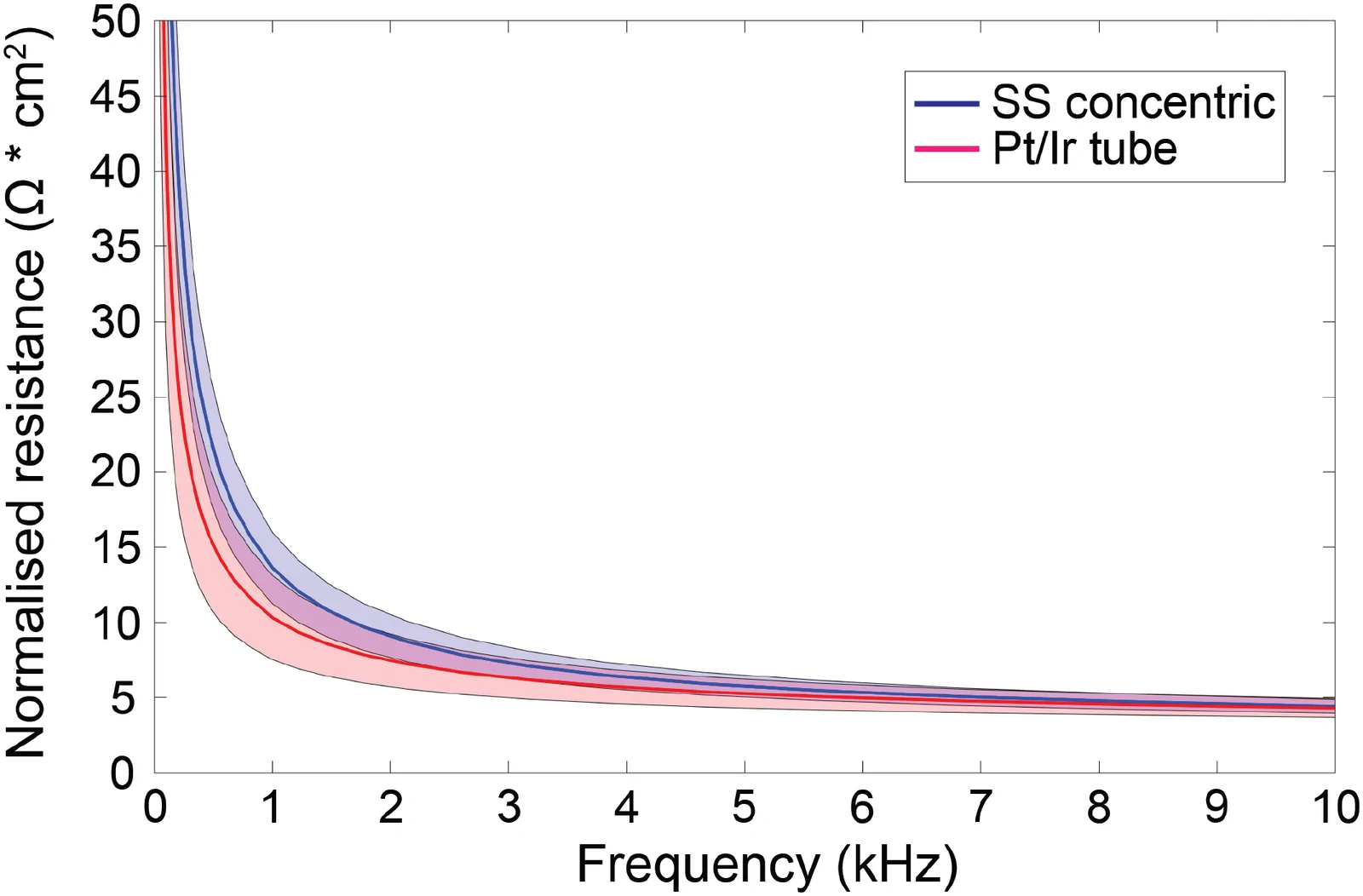
Electrical impedance spectroscopy (EIS) of Pt/Ir *vs* stainless steel (SS) electrodes used by BaDGE probe, normalized to respective electrode surface areas (average surface area of distal and proximal electrodes is used in the concentric SS case). EIS performed on 4 Pt tube BaDGE probes *vs* 4 concentric SS needle BaDGE probes, with means and SEM ranges shown.

Given that the electric pulses are not delivered at a single fixed frequency but instead consist of a single capacitive discharge into tissue, we decided that a more relevant comparison would be the voltage drop across the electrode–electrolyte interface for both GET electrode types. Voltage maps were obtained for n = 4 probes for each of the SS and Pt/Ir electrode types, by sampling the voltage responses in immediate proximity to the electrodes immersed in 0.9% saline. For each map, 26 points were sampled with a 225 μm diameter SS recording electrode, over an X range of 1800 μm with a resolution of 150 μm, using 13 points offset by 150 μm from the electrode. The recording electrode was mounted on a T-LS13-M motorized XY stage (Zaber Technologies, Canada), and movement between points was randomized for each electric potential map. At each point, a full discharge from 200 V (across the two electrodes, with a distant ground) was recorded, though the peak voltage was of particular interest. Figure 9(a) shows the field mapping procedure, with recording positions along the X and Y axes. For each X position, a voltage ratio was calculated according to the peak voltage at the Y = −150 μm position divided by the peak voltage at the Y = 0 μm position (touching the electrode). Figure 9(b) shows the mean voltage ratio (and SEM) for both GET probes across the inner (distal) and outer (proximal) electrodes. From the figure and subsequent statistical analysis, it can be observed that in general, there is no statistical difference in the voltage drop ratios between the Pt/Ir tube electrode pairs *vs* the SS concentric electrode pairs. Both probe types exhibit a higher voltage drop toward the null point between the two electrodes (separated by 1 mm). The Pt/Ir tube electrodes have a generally symmetrical voltage drop between the distal and proximal electrodes, but the SS concentric needle probe exhibits a larger voltage drop across the length of the inner needle (which has a smaller diameter) compared to the outer needle.

**FIG. 9.**
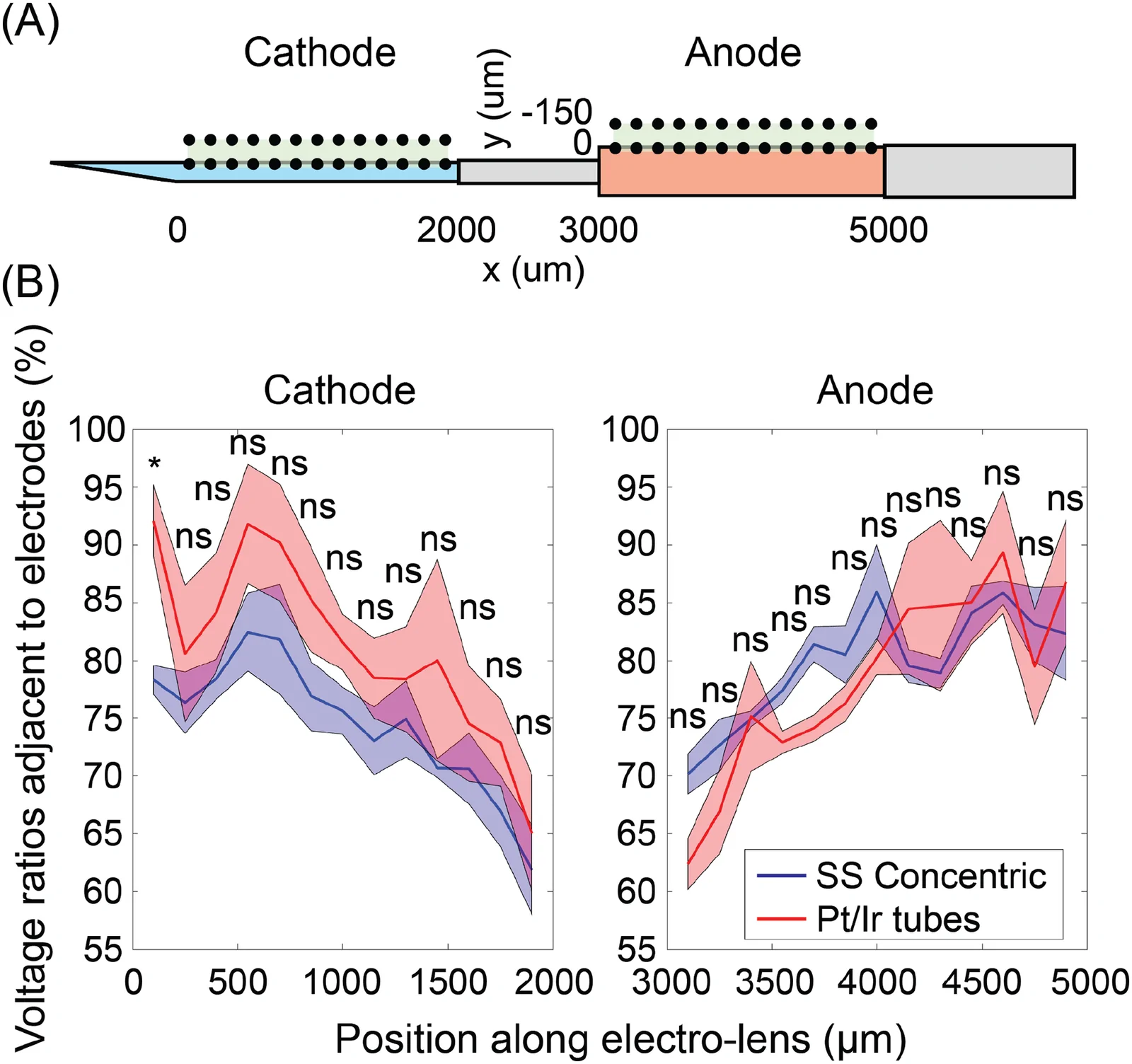
Demonstration of comparability of the use of SS concentric needle-type BaDGE electro-lens probe with an electro-lens fabricated from Pt/Ir tube electrode elements, measured as voltage ratios across the BaDGE probe electrode–electrolyte interface for 50 mA current pulses in 0.9% saline solution. (a) Voltage mapping within the electric field was performed on and immediately proximal (150 μm) to the BaDGE electro-lens. (b) Mean voltage drops (and SEM) across electrode–electrolyte interface of Pt/Ir tube electro-lens constructs (n = 4) *vs* SS concentric needle (n = 4) electro-lens probes, measured via voltage mapping along and adjacent to electrodes. Comparison of values was performed using an ordinary two-way ANOVA with Holm–Sidak multiple pairwise *post hoc* comparisons. D'Agostino–Pearson omnibus normality test passed (*p* = 0.0772). For pairwise comparisons between material types at each position, ns indicates no significant difference (*p* > 0.05), * *p* ≤ 0.05. Note that for all probes, a 1 mm separation exists between the two 2 mm longitudinally extended electrode elements, reflecting the electro-lens tuner element.

A summary of the key engineering specifications of the final fully automated single capacitive discharge BaDGE GET probe is shown in Table II. The table includes specifications of the needles/electrodes, capacitor, typical pulse delivery parameters, and data obtained during characterization studies including dead space volume and discharge reliability.

**TABLE II.** Key engineering specifications of the final fully automated single capacitive discharge BaDGE GET probe design.

Parameter	Value
**Needle and electrode dimensions**	
Outer needle (proximal)	28-gauge stainless steel (0.362 mm OD/0.267 mm ID)
Inner needle (distal)	34-gauge stainless steel (0.184 mm OD/0.083 mm ID)
Internal separation	42 *μ*m
Exposed electrode length	2 mm per electrode
Electro-lens tuning gap	1 mm separation between electrodes
Dielectric insulation	∼5 *μ*m vapor-deposited parylene-C (1.1 kV breakdown)
**Internal electronics**	
Capacitor type	Multilayer ceramic capacitor
Capacitance	2.2 *μ*F
Maximum voltage rating	450 V
**Delivery and discharge properties**	
Typical charging voltage	120–200 V
Pulse waveform	Single exponentially decaying pulse
Discharge time constant	Depends on tissue resistance (e.g., 1.4–51.2 ms demonstrated in vitro)
System fluid dead space	13.2 *μ*l ± 4.01 (mean ± SEM)
Automated discharge reliability	94.14%

## VI. *IN VITRO* VALIDATION OF WIRELESS CONCENTRIC NEEDLE BaDGE PROBES

We validated the effectiveness of the single capacitive discharge SS concentric needle BaDGE GET probe using the *in vitro* HEK293 cell model with GFP reporter plasmid DNA delivery. This study considered the effect of manipulating the discharge time constant by controlling the local resistivity using a sucrose carrier (conductivity-clamping, after Ref. 3). This was accomplished by adjusting the ratio of 0.9% phosphate-buffered saline (PBS) and 10% sucrose within the suspension solution. Sucrose is a disaccharide that is not transported directly into cells and does not affect metabolism. Both solutions are isotonic (300 mOsm); hence, any resulting mixture maintains physiological osmolarity, which allowed us to decrease the solution conductivity by reducing the proportion of PBS to sucrose. Figure 10 shows the relationship between the ratio of PBS to sucrose *vs* the average decay time constant of a discharge (linear and log –log plots). Discharges were produced by discharging a 2.2 μF capacitor charged to 200 V through a BaDGE probe (without hub electronics) immersed in 200 μl of each solution. Also shown are the time constants from subsequent *in vitro* experiments in which each solution included 0.5 mg/ml of GFP reporter plasmid DNA under a cytomegalovirus promoter and incorporating a nuclear localization signal to aid quantification of cell transfection (CMVp-hrGFPnls).

**FIG. 10.**
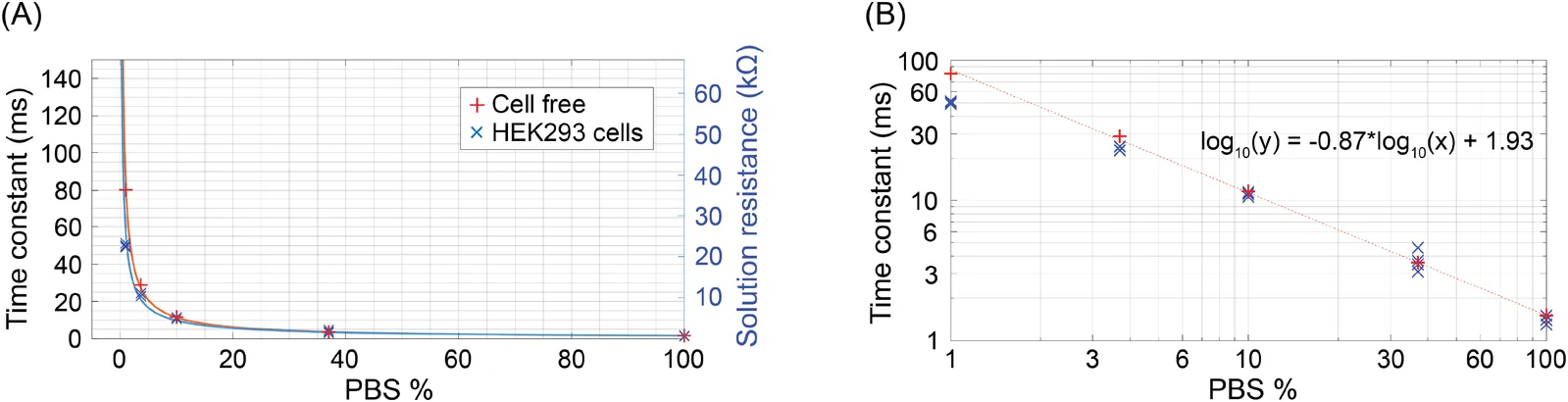
Comparison of discharge time constants as a function of resistivity by varying the PBS to sucrose ratio for single capacitive discharge SS concentric needle BaDGE GET probe. (a) Exponential relationship exists between PBS percentage and decay time constant. Red markers denote the average time constants (with insignificant variability) found in solutions with varying PBS concentrations diluting the 10% sucrose carrier, while blue markers show time constants found during transfection experiments (n = 4), with solutions also containing DNA and fully covering a monolayer of HEK293 cells. Equivalent solution resistance [right axis (a)] was calculated based on R = τ/C where C = 2.2 μF. (b) Linear fitting on the log_10_–log_10_ relationship between PBS % *vs* time constant allows for fitting of the exponential relationship in (a). The R^2^ value for the linear regression on the log_10_–log_10_ data for the no cells scenario is 0.999, with the equation shown on the plot. All measurements utilized a 200 V capacitor charging voltage.

On the day of transfection, five solutions were prepared, with PBS percentages of 100%, 37%, 10%, 3.7%, and 1%. The GFP reporter DNA was added to each solution at 0.5 mg/ml. For each transfection, an 18 mm round coverslip seeded with a monolayer of HEK293 cells ( ∼ 80% confluence) was taken from incubation media and placed onto the GET platform, with the concentric needles of the BaDGE probe positioned just above the cells via micromanipulator. 200 μl of DNA in PBS/sucrose was then pipetted on top, and a single capacitive discharge (2.2 μF) at 200 V was performed. The coverslip was then removed and placed in fresh media and incubated for 3 days until imaging. Four coverslips for each of the five solutions were transfected, with an additional three coverslips (all with 100% PBS) used as controls. The same probe was used for all 23 coverslips.

After 3 days, native GFP fluorescence signal within the HEK293 cell nuclei was confirmed using a LSM 710 confocal laser scanning microscope (Zeiss, Germany) with a 5×/0.16 objective and argon ion 488 nm laser. The GFP signal was then amplified using an immunofluorescence protocol (see Methods). Image fields of 2048 × 2048 pixels were stitched together to produce complete images of the transfection area for each coverslip. Figure 11 shows an example image from each PBS/sucrose/DNA combination. An outer and inner area of GFP expression was manually segmented for each image to encompass the maximum area in which transfection was detected, and the area where cell loss occurred (due to a combination of high electric field strengths, gassing, and mechanical disruption).

**FIG. 11.**
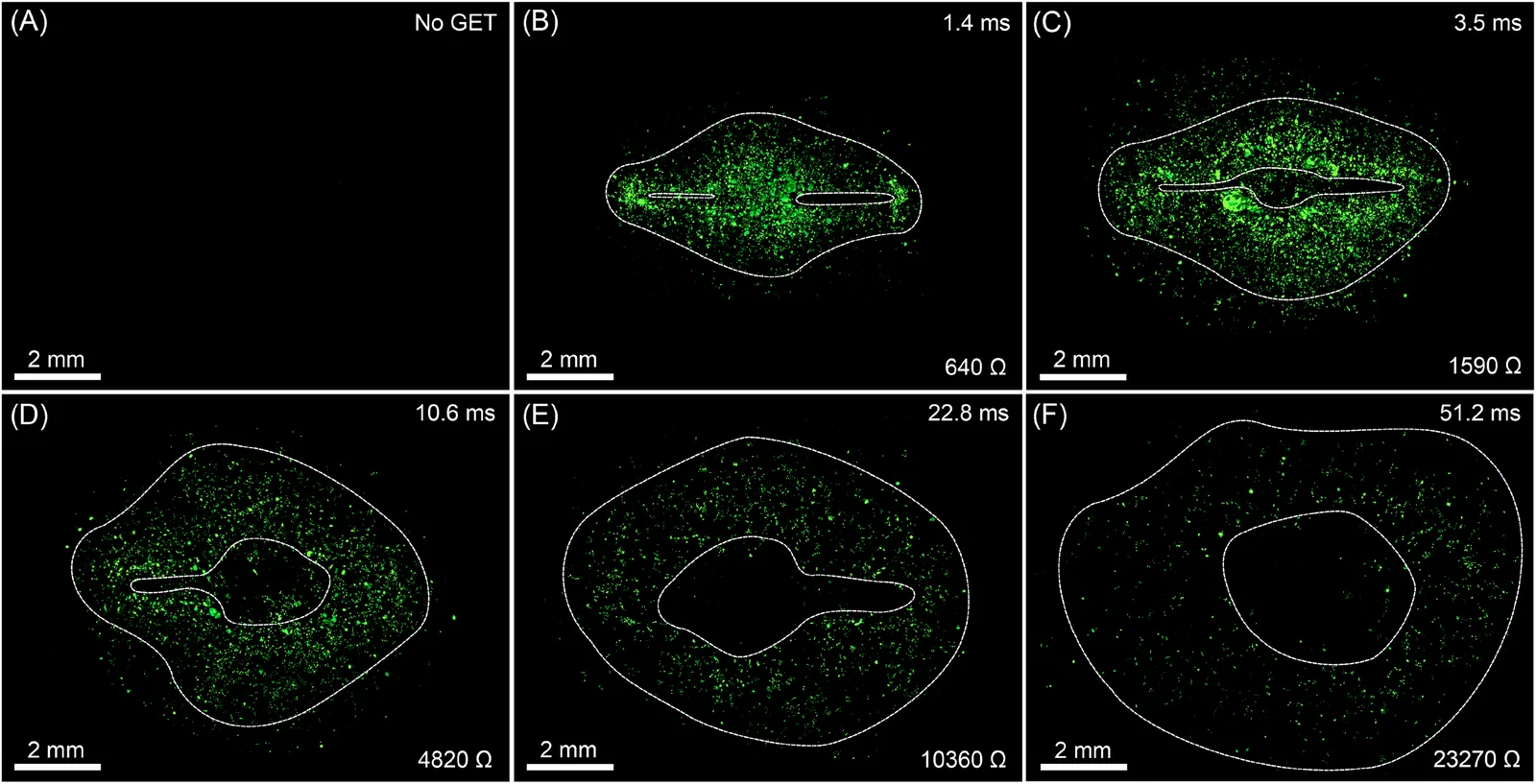
Representative images of SS concentric needle BaDGE GET probe-based transfection of HEK293 cells with plasmid GFP-encoding DNA solutions with increasing resistivities. (a)–(f) Ratios of 0.9% PBS:10% sucrose: (a) control (no GET) 100:0; (b) 100:0; (c) 37:67; (d) 10:90; (e) 3.7:94.3; (f) 1:99. All conditions used a 2.2 μF capacitor charged to 200 V. The resulting (averaged, n = 4 per group) discharge time constants are indicated along with calculated resistance across the electro-lens. These data show that increasing extracellular resistivity expands the spatial volume of transfection, while reducing transfected cell density. Dotted lines delineate contiguous areas in which detectable GFP reporter gene expression has occurred; internal spaces likely reflect cell loss due to field strength and electrode gassing displacing the HEK293 cell monolayer. Confocal fluorescence imaging 3 days after GET.

The images were then processed using a cell counting script in MATLAB to segment out individual cells using morphological operations and watershed transforms. Figure 12(a) shows boxplots for the number of transfected cells for each GET configuration (five PBS percentages and a control for the GFP DNA carrier solution; ranging from 100% of 0.9% PBS: 0% of 10% sucrose, to 1% of 0.9% PBS: 99% of 10% sucrose). Statistical analysis utilized one-way ANOVA with *post hoc* Holm–Sidak pair-wise comparisons between each transfection configuration (Prism, GraphPad Inc, USA). To compare transfection areas, the areas contained within the outer and inner perimeters in each image (as seen in Fig. 11) were integrated, with a scaling conversion of 1.42 μm^2^ per pixel. The area within the outer perimeter was considered the “total” area encompassing the maximum extent where transfection could be detected, while the inner perimeter denoted the “null zone” area, where cell density was impacted. Figure 12(b) shows boxplots of these two areas with individual data overlay for each configuration, indicating an inverse trend between PBS percentage and total/null zone areas. This was also apparent by study of the maximum radius from the BaDGE GET probe (perpendicularly) for transfection. The maximum transfection radii and null zone radii were calculated by taking the maximum distance between the centroid and upper/lower boundaries of the total and null zone areas, respectively [Fig. 12(c)].

**FIG. 12.**
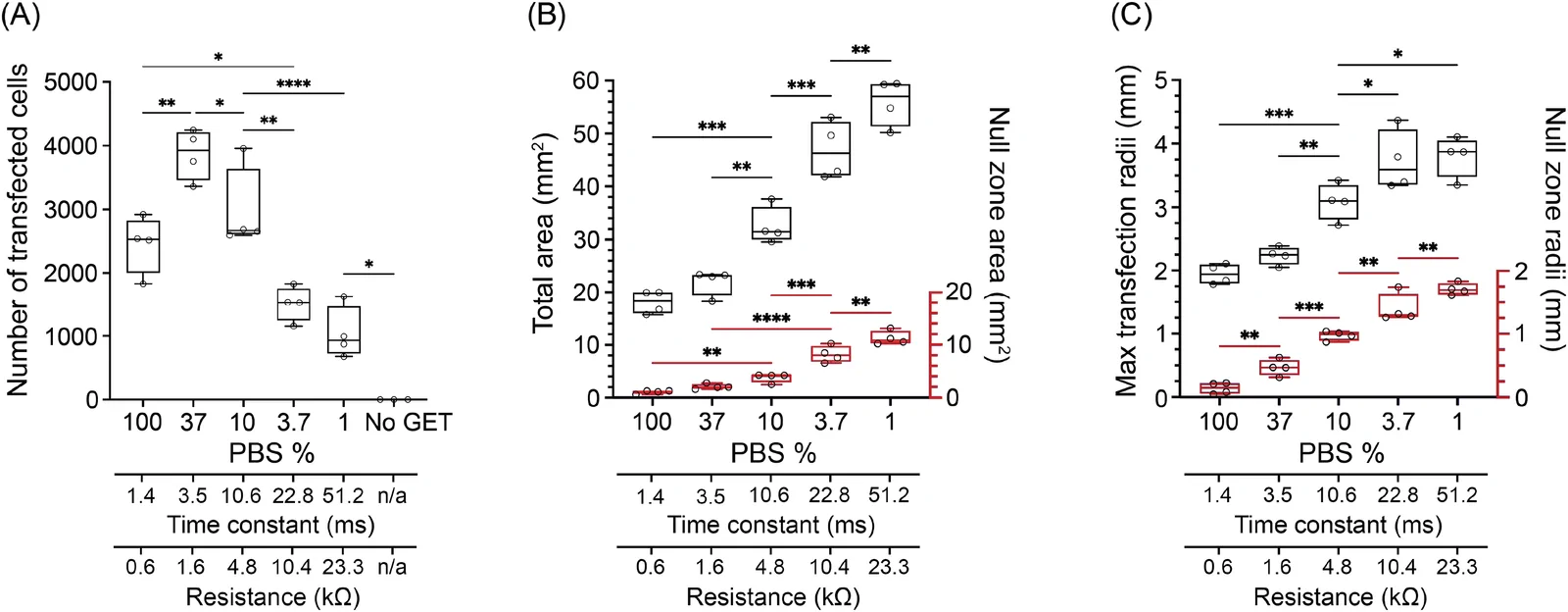
Effect of PBS:sucrose ratio (defining extracellular local resistivity) on single capacitive discharge HEK293 cell GET for GFP reporter plasmid DNA. (a) Number of cells transfected using 200 V, 2.2 μF capacitor discharge, with X axis showing average time constant and equivalent resistance across electro-lens for each PBS% group. (b) The surface area of GFP-positive cells based on the outer perimeter as shown in Fig. 11. Inner “null zone” area is shown in red. (c) The maximum radii of successful transfection and (in red) maximum “null zone” radii perpendicular to the axis of the probe. N = 4 per group. Boxplots show first quartile, median, and third quartile from each data set. *, **, ***, and **** signify *p* < 0.05, 0.01, 0.001, and 0.0001 respectively (one-way ANOVA with Holm–Sidak multiple pairwise *post hoc* comparisons). Individual data are overlaid.

## VII. *IN VIVO* REPORTER EXPRESSION IN MOUSE SKELETAL MUSCLE

The efficacy of spatially controlled vector-free DNA delivery to skeletal muscle via the BaDGE electro-lens SCD-CC-GET probe was investigated *in vivo* through a long-term mouse hindlimb muscle study. Under a UNSW Animal Care & Ethics Committee approved protocol, CMVp-mCherry reporter plasmid DNA [4485 bp; after;^3^ 2 μg/ μl 50 μl; in 10% sucrose (no PBS) for CC] was injected into the left gastrocnemius muscle of an adult mouse using the BaDGE GET probe, under isoflurane (1.5% in O_2_) anesthesia. This was followed immediately by capacitor discharge (264 μC) to achieve instant GET to the muscle fiber sarcolemma. The mouse was euthanized 18 months later and the native mCherry fluorescence was imaged after PFA fixation by confocal microscopy of cryosectioned sections, demonstrating extensive expression within the hindlimb muscle fascicles (Fig. 13).

**FIG. 13.**
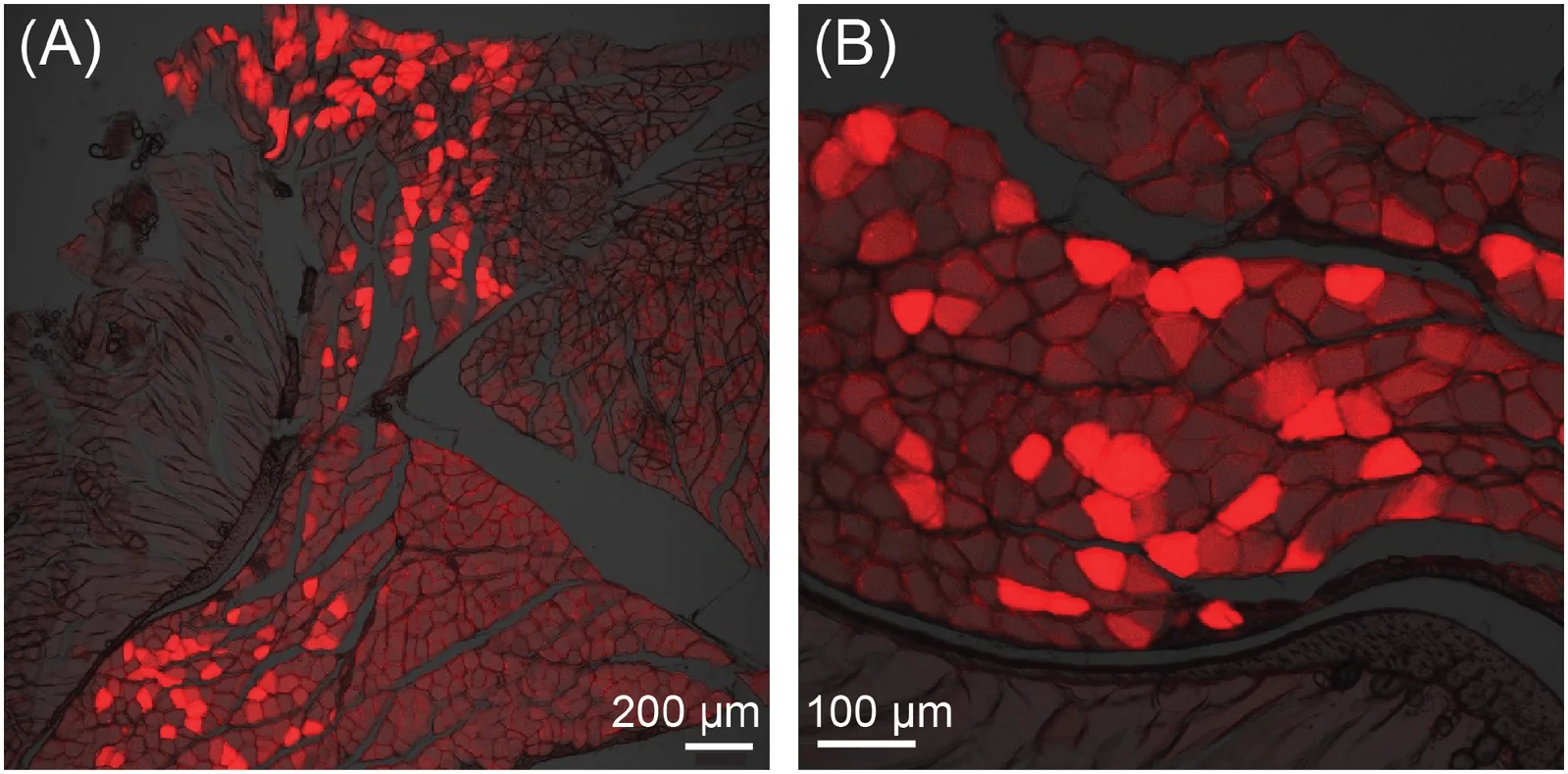
Stable long-term *in vivo* expression of mCherry reporter plasmid DNA delivered to the mouse gastrocnemius muscle by BaDGE GET probe using SCD‐CC‐GET (2.2 μF capacitor, 120 V; CMVp‐mCherry plasmid DNA (50 μl; 2 μg/ μl; 10% sucrose carrier) injected at the tip of the electro-lens via the fluidic channel). Native mCherry fluorescence overlaid on transmitted light channel. Tissue from animal euthanized 18 months post‐GET (50 μm cryosections; 561 nm Ex, 567–633 nm Em).

## VIII. DISCUSSION AND FUTURE DEVELOPMENT

### A. Device architecture and pulse delivery

A new BaDGE electro-lens GET probe was developed to provide a compact and wireless vector-free DNA delivery. This GET probe featured a linear array of two 2 mm conductive electrode surfaces at the distal ends of two concentric parylene-coated SS needles ( ∼ 30 μm internal separation) with a 1 mm insulated electro-lens element to provide a highly focused electric field for close-field GET. These dimensions were chosen to balance optimum electric field physics with the practical constraints of deep tissue targeting. Our modeling established that the predicted transfection zone was maximized with a tuning element of 1 mm compared to shorter distances, while greater distances (e.g., 4 mm) would lead to discontinuous zones resulting in poorer transfection. This total array length of 5 mm was also well aligned with *in vivo* validation, such as for targeting of gastrocnemius muscles or subcortical brain structures in rodent models. The practical manufacturing of the array is equally complex regardless of tuning element length; so, the technology can be easily adapted for smaller or larger targets. This indicates that the electro-lens technology is well suited for personalized nucleic acid therapeutics where gene therapy can be limited to the needed tissue target volume. For example, this is well aligned with the potential for solid tumor DNA/RNA vaccine applications.

Compared with current electrode arrangements, featuring a set of plate electrodes, or several needle electrodes,^18^ this GET probe requires only a single penetration, with the concentric needles providing both the fluid delivery and (single) pulsed electric field for GET. Hence, the GET probe is not only minimally invasive compared to current methods, but the “electro-lens” enables precision spatiotemporal control of gene delivery within predetermined tissue volumes. The use of SS as the electrode material, which was necessary for the concentric design, was also shown to have comparable utility to Pt/Ir with respect to voltage loss across the electrode–electrolyte interface and hence was deemed a suitable substitute. Plate and needle electrodes used for electroporation are frequently made of SS (AISI 304 or 316) or titanium,^18^ so the choice of material for the GET probe is not unusual.

A further distinguishing technical feature of the BaDGE GET probe was the onboard charging and discharge mechanism for providing delivery of the DNA without a connection to an external pulse generator. Conventional electroporation systems rely on bulky tethered external generators in order to deliver currents of up to an amp via square-wave pulse trains into the target tissue to achieve hundreds of volts across separated electrodes operating against standard tissue conductivity. Our GET probe integrates the discharge mechanism into the syringe housing sleeve in such a way that the discharge is automatic upon full depression of the syringe plunger, which triggers a copper switch and completes the circuit between the capacitor and electrodes. Intrinsically, the unprecedented efficiency of this electro-lens-based compression of the local electric field enables efficient gene expression with a minimal single capacitive discharge (SCD). A typical pulse protocol for an open-field electroporator of 3 × 52 ms pulses at 500 mA (e.g., CELLECTRA^25^) delivers 78 mC of charge, which is heavily associated with irreversible electroporation, Joule heating, and potential tissue damage. By contrast, under the highest settings used in our study, a 2.2 μF capacitor charged to 200 V transfers a mere 440 μC of charge, which is 177 times less than that of the aforementioned typical system. Additionally, the maximum total energy delivered by a single discharge (where $E=\frac{1}{2}CV^{2}$) is only 0.044 J. Even if all of the energy stored in the capacitor was transmitted as thermal energy, this amount of energy output is negligible and will not induce noticeable Joule heating or thermal damage in the tissue. Overall, the BaDGE GET probe, through the use of single capacitive discharge and conductivity-clamping, has been demonstrated here to be able to effectively achieve high transfection efficiency, while also being wireless, compact, and attachable to any commercially available Luer Lock syringe, with essentially zero additional dead space to the delivery system.

### B. Biological validation

The BaDGE probe in this study was also shown to be highly capable of efficient transfection of HEK293 cells (Fig. 11), with the transfection efficiency being highly dependent on the immersion solution resistivity according to its PBS and 10% sucrose composition. From a 100:0 PBS:sucrose ratio, to a 1:99 ratio, the average time constant of discharge increased from 1.4 to 51.2 ms, corresponding to a resistance increase from 0.6 to 23.3 k Ω (as the capacitor was 2.2 μF). This had the effect of significantly increasing the size of the field of gene expression. Figure 12 shows how the total area of detected GFP reporter expression increases as the solution resistivity increases due to increasing sucrose. However, the null zone area (and radius) also increases correspondingly, which is attributable to factors including mechanical disruption from gassing and potentially from irreversible electroporation and thermal (caloric heating) effects at very high field strengths.^20^ The spatial variance in density of transfected HEK293 cells within the GET electric field threshold boundary are likely to reflect model conditions, as indicated, as well as potential distortion of the electric field due to the surface tension fluid meniscus effect around the needle probe. Nevertheless, this study confirmed that conductivity-clamping effectively boosts localized electric field strengths, allowing the single capacitive discharge to achieve large transfection zones.

The efficacy of the BaDGE GET system was also investigated through an *in vivo* longitudinal mouse hindlimb study. SCD-CC-GET delivery of a CMVp-mCherry reporter plasmid into the gastrocnemius muscle resulted in robust and stable expression that was maintained even up to 18 months after the procedure (Fig. 13). Importantly, the image shows that normal muscle fascicle histology was well preserved even at 18 months, which demonstrates the safety of the system and procedures, unlike the necrotic tissue damage that can result from conventional electroporation techniques. Significantly, the safety of conductivity-clamped gene electrotransfer has also recently been validated in a human clinical trial.^27^ Although this longitudinal *in vivo* study provides a robust proof of concept for the SCD-CC-GET delivery mechanism in a live animal model, we acknowledge that it represents a single demonstration. Comprehensive *in vivo* studies in various animal models to assess transfection efficiency and tissue safety concerns will be needed before the findings in this study can be generalized to broader therapeutic applications.

### C. Translational considerations

There are a number of factors to be considered before the technology described in this study can be scaled up for mass manufacture and translated to clinical usage. From a manufacturing point of view, the material choice and construction of the needle array should be considered. Our current technology utilizes surgical-grade stainless steel needles insulated via vapor-deposited parylene-C, with one needle inserted concentrically within the other. Although the process has been highly refined, it represents the largest bottleneck, because of the reliance on an external facility, at high cost. Assembly of the concentric needles requires particular care to ensure that the 5 μm insulating coating is not damaged. The attachment of the stainless steel needles onto the PCBs is also a manual process that requires either a specialized solder, or snap-fit spring fittings. For future development of this technology in preparation for mass manufacture, an exploration of biocompatible materials such as aluminum should be conducted. Aluminum needles can be hard anodized quickly to form an insulated layer, allowing for bulk insulation of needles to increase production speed and reduce per unit costs.

Sterility of the components is another factor relevant to clinical usage. The inherently modular design of the BaDGE GET system offers advantages in this regard. Because the needle hub assembly is a separate component to the automated discharge mechanism contained within the sleeve assembly, these parts can be manufactured, sterilized, and packaged separately. The syringes are of course packaged sterile as well. During usage, the clinician will take the sterile syringe from the package and load the DNA solution into it. Only after this will the two components of the BaDGE device be taken out of their sterile packaging and attached to the syringe. The needle guard, which is attached to the needle hub, and which has also been sterilized, protects the needles as the device is charged. When the device has been charged, the needle guard is removed, and immediately the needle is inserted into the patient, minimizing the exposure time to potential pathogens.

Finally, regulatory considerations should also be considered. Our BaDGE system relies on a comparatively simple discharging system, featuring only passive components, no software, and no high voltage/current circuitry. Although the initial voltage (120–200 V) is high, a typical discharge is complete within 5–10 ms and represents a fixed charge, a point of differentiation from conventional current or voltage-controlled electroporation systems. As such, we anticipate that obtaining device safety approval through the regulatory pathway should be significantly less complicated compared to conventional electroporation systems.

### D. Current limitations

While the SCD-CC-GET platform described in the present study successfully demonstrates wireless gene delivery and transfection of plasmid DNA to cellular and animal models, several limitations still need to be addressed in future developments.

The finite element computational modeling in COMSOL preformed in Sec. II intentionally utilized a simplified homogeneous model with a globally set 1 S/m tissue conductivity. This was because the intention was to analyze the tuning of the electro-lens element geometry on the resulting electric field profile. The actual conductivity in tissue would be more heterogeneous and anisotropic, and so these simulations do not predict the absolute transfection volume that would be achieved *in vivo*. A more rigorous computational modeling study using this electrode geometry layout has been performed in our prior work,^23^ in which we model the anisotropic conductivity within human brain structures and simulate electric field profiles and predict gene transfection efficacy.

Another limitation of the technology itself is that the discharge profile of the SCD pulse is dependent on the target tissue and cannot be strictly controlled. The capacitor and delivery voltage is fixed, so the total charge delivered ( Q=CV) is known and constant; however, the discharge time constant ( τ=RC) is dependent on the total system resistance of the target. Our conductivity-clamping utilizes this to increase the time constant by artificially increasing the target resistance, as demonstrated in earlier studies^3,28^ as well as in the present study, where it enables a fixed charge delivery to have increased efficiency at transferring the polyanionic DNA/RNA species to the plasma membrane. While our working baseline assumes a tissue resistance of approximately 2 kΩ with conductivity-clamping, the reality is that variability in clamping efficiency of extracellular tissue conductivity (e.g., due to connective tissue or fat deposits) will impact the resulting efficiency of gene transfection. Application of this electro-lens gene transfer technology in different therapeutic indications will therefore require appropriate *in vivo* validation.

Because our BaDGE GET probe is specifically designed for single use, and is thus disposable, we believe that long-term durability of the hardware is not a major concern. There is, however, a risk of user error due to the assembly of the modular parts, such as due to misalignment, which may result in the syringe plunger failing to activate the copper switch at the end of its travel. Future designs will increase the keying between assembly components to make it impossible to incorrectly align parts.

## IX. CONCLUSION

A single concentric needle system housed in a compact hub incorporating a capacitor and switch was designed and manufactured to provide a GET probe allowing for the delivery of DNA transgenes. The intrinsic fluidics delivered the DNA in a carrier solution designed to reduce local tissue conductance, thereby boosting the electric field strength achieved by the discharge of the capacitor. Given that the translocation of the polyanionic DNA (or mRNA) to the cell plasma membrane requires a minimum electric field strength, this “conductivity-clamping,” demonstrated by the effect of serial dilutions of saline with 10% sucrose in the carrier, proved highly effective at enhancing the transfection volume for the fixed charge, where the boundary of the GFP fluorescence positive cells was a biomarker for this electric field threshold. The modeling and *in vitro* validation of spatiotemporal control of GET were found to be highly translatable to the delivery of naked mCherry reporter-encoding DNA to mouse hindlimb muscle fibers, where safety and efficient long-term expression were confirmed.

## X. METHODS

As the engineering development and validation of our technology are presented in an integrated format throughout the manuscript, Sec. X A–10 F provides a concise consolidated summary of the principal experimental methodology.

### A. Computational modeling

Computational models were developed in COMSOL Multiphysics 5.5 (COMSOL, Sweden). Electrodes were modeled as cylinders with lengths of 2 mm, diameter of 400 μm, and separation distances of 0.2, 0.4, 1, and 4 mm. Both the electrodes and surrounding volume were meshed using tetrahedrals with a maximum element size of 100 μm. Stationary electric currents physics was applied to the model with the surrounding volume set to a conductivity of 1 S/m and zero current flux at the boundaries. The outer surface of one electrode was set as the ground reference while the other was set to 8 V. Electric potential and electric fields in the stimulation volume were simulated for each condition.

### B. Concentric needle probe manufacturing

Needle assemblies were manufactured by first insulating a 51.5 mm 34-gauge needle and 45 mm 28-gauge needle with 5 μ of parylene-C coating, using vapor deposition. The 34-gauge needle was then threaded through the 28-gauge needle, protruding by 3 mm at the cutting edge, and fixed in place with polyurethane. 2 mm of insulation was then etched away from the distal end of each concentric needle using an 193 nm excimer laser, leaving an insulated gap of 1 mm between the two uninsulated “electrodes.” The needle assembly was then attached to a PCB via a set of spring contacts for the outer 28-gauge needle and a press-fit receptacle for the inner 34-gauge needle. This PCB, featuring contact pads for charging, was inserted into a zero-dead space custom hub.

To complete the system, a syringe was placed inside a three-part sleeve, to which the hub housing the needle assembly was connected. Within the sleeve, a copper switch was created using a set of copper pads and springs, which are electrically connected to a capacitor and to the PCB in the needle hub via connector pins. When the syringe plunger is fully depressed, the copper switch circuit is closed, causing each side of the capacitor to be electrically connected to the two electrodes, producing a discharge in the target.

### C. Engineering characterization

Electrical discharge efficacy and accuracy were evaluated by monitoring the capacitor voltages using a DS1074 oscilloscope. 1k resistors were coupled to the electrodes, and the capacitors (2.2 μF) were repeatedly charged to 30 V via an external power supply, and discharged across the test resistor. Noise profiles were monitored, and the discharge time constants were recorded, with an expected value of 2.2 ms. In total, 54 trials were conducted across 5 probes.

To compare the impedance of stainless steel electrodes to Pt/Ir, electrical impedance spectroscopy was performed. SS and Pt/Ir GET probes (with paired electrodes) were suspended in 100 ml of 0.9% saline, with EIS performed using a CHI604E electrochemical analyzer. 12 points were obtained per decade, between 1 and 100 000 Hz. Recorded impedances were normalized according to electrode surface area.

Voltage loss at the electrode–electrolyte interfaces was measured using a recording electrode mounted on a T-LS13-M motorized XY stage. Electrodes were immersed in 0.9% saline. The recording electrode sampled 13 points along each electrode, touching the electrode, with a resolution of 150 μm, and 13 points parallel to each electrode offset by 150 μm. At each recording site, a full exponential decay discharge from 200 V was produced across the electrodes, and the resulting voltage profile was recorded. At each location along the electrodes, the voltage loss across the bilayer was calculated by comparing the peak voltage at 150 μm offset from the electrode with the peak voltage touching the electrode. 4 pairs of electrodes for both the SS and Pt/Ir types were evaluated.

### D. *In vitro* validation

HEK293 cells were cultured in Dulbecco's Modified Eagle Medium (DMEM) with 5% Fetal Bovine Serum (FBS) and 1% penicillin-streptomycin culture media according to standard cell culture protocols. GFP reporter plasmid DNA (CMVp-hrGFPnls) was prepared according to standard protocols using the Qiagen maxiprep kits. Plasmid DNA was suspended in mixtures of standard PBS (0.9% NaCl) and 10% sucrose solution, with ratios of 100%, 37%, 10%, 3.7%, or 1% PBS (vs sucrose). For each set of transfections, a BaDGE GET probe was rested on an 18 mm round coverslip seeded with a monolayer of HEK293 cells. 200 μl of the PBS/sucrose solution, containing GFP plasmid DNA at a concentration of 0.5 mg/ml, was then pipetted onto the coverslip, fully immersing the electrodes. The 2.2 μF capacitor in the GET probe, charged to 200 V, was then discharged across the electrodes to transfect the cells.

After the coverslips were incubated in a CO_2_ incubator for 3 days, the cells on the coverslips were fixed in 4% paraformaldyhyde for 15 min, then permeabilized with 0.1% Triton X100 in 4% donkey serum, incubated with 1:500 rabbit - anti GFP antibody in 0.1% Triton overnight, incubated with Alexa488-conjugated donkey anti-rabbit IgG H&L secondary antibody in 0.1% for 4 h, before being mounted. These mounted coverslips were imaged using a LSM 710 confocal laser scanning microscope with a 5×/0.16 objective and argon ion 488 laser.

### E. *In vivo* validation

Validation in a long-term mouse model was performed by injecting 50 μl of CMVp-mCherry plasmid DNA suspended in 10% sucrose at 2 μg/ μl into the left gastrocnemius muscle through the concentric needle BaDGE GET probe. The mouse was anesthetized using isoflurane (1.5%) under standard operating procedures. Immediately after delivery of the plasmid DNA solution, instantaneous gene electrotransfer to the muscle fibers was achieved by discharging the capacitor coupled to the electro-lens probe (2.2 μF at 120 V). The mouse was euthanized 18 months later via injection of sodium pentobarbital according to standard procedures. The mouse was then fixed using PFA fixation, and then the gastrocnemius muscle was cryosectioned (50 μm). Sections were imaged using the LSM 710 confocal laser scanning microscope for mCherry fluorescence (61 nm Ex, 567–633 nm Em).

### F. Statistical analysis

Statistical analyses were performed using GraphPad Prism 11. One-way and two-way analysis of variance (ANOVA) tests were performed after validating normal distribution of the data using the D'Agostino–Pearson omnibus normality test. Holm–Sidak multiple pairwise post hoc comparisons were then undertaken to evaluate individual factors. Relationships were deemed significant for *p* ≤ 0.05. Mean values reported are presented with ± standard error of the mean (SEM).

## Data Availability

The data that support the findings of this study are available within the article.
